# Covid re-emergence: Do we have to mask up again?

**DOI:** 10.1016/j.amsu.2022.104539

**Published:** 2022-09-02

**Authors:** Jawad Basit, Sumia Fatima, Usman Ali Akbar, Omaima asif, Mohammad Ebad ur Rehman, Sajeel Saeed, Ka Yiu Lee

**Affiliations:** Department of Medicine, Rawalpindi Medical University, Pakistan; Northwell Health, Department of Internal Medicine, New York, USA; Department of Medical Education, Rawalpindi Medical University, Pakistan; Department of Medicine, Rawalpindi Medical University, Pakistan; Swedish Winter Sports Research Centre, Department of Health Sciences, Mid Sweden University, Sweden

In 2020, the SARS-COV2 variant of the Corona Virus wreaked havoc and sparked an unprecedented pandemic. It destroyed the economies of several countries and altered the global health situation. High-risk groups included older adults and people with co-morbidities such as diabetes, cardiovascular disease, and malignancies [1]. The COVID-19 pandemic seriously affected industrial sectors, especially agriculture, petroleum, and manufacturing [2]. The Coronavirus disease is caused by the Sars-Cov-2 and presents mainly with mild symptoms such as dry cough and fever but sometimes progresses to severe pneumonia and results in Acute Respiratory Distress Syndrome (ARDS) [3]. As of August 5, 2022, there have been 579 million confirmed cases and 6.4 million deaths globally [4].

Even though the world has yet to recover from the shock fully, danger still exists. As cases reached a two-month high, South Korea's PM issued a COVID outbreak warning [5]. From May to July 2022, countries like Malaysia, the United Kingdom, the United States of America, Greece, Ireland, and Singapore reported an immense increase in Covid-19 related hospitalizations with relative changes of +129%,+61%,+200%,+133%,+72%, and +175% respectively [6].

Although Observational data suggest that the risk of severe disease with Omicron infection is lower than with other variants, Omicron sub-variants like BA.4 and BA.5 are predicted to cause many COVID-19 cases since the coronavirus is mutating rapidly. As of August 2022, The United States is currently reporting more than 130,000 cases per day [7]. According to the data available by June 2022, about 35% of cases in the United States were of BA.4 and BA.5, with BA.5, the most transmissible variant of the coronavirus, making up to 23.5% and BA.4 accounting for 11.4% [8].

According to the Portuguese National Institute of Health, by May 2022, BA.5 was responsible for almost 37% of positive cases in their country [9]. Like South Africa's previously stated 12% daily growth advantage, the anticipated daily growth advantage for BA.5 over BA.2 is 13%. BA.5 will overtake all other variants in Portugal, assuming this growth rate continues [9].

Recent studies reveal that the BA.2.12.1, BA.4, and BA.5 sub-variants significantly avoid neutralizing antibodies elicited by vaccination and infection [10]. Furthermore, neutralizing antibody titers against the BA.4, BA.5, and BA.2.12.1 sub-variants were lower than those against the BA.1 and BA.2 sub-variants, indicating that the SARS-CoV-2 omicron variant has continued to evolve with increasing neutralization escape [10]. This data provide meaningful immunologic background for the present surges induced by the BA.2.12.1, BA.4, and BA.5 sub-variants in populations with high vaccination rates and prior BA.1 or BA.2 infection [10].

The overall proportion of BA.4 and BA.5 in the European Union/European Economic Area is currently low. Still, the high growth advantages reported, suggest that these variants will become dominant in the EU/EEA in the coming months [9].

The Scripps Research Translational Institute director, Dr. Eric Topol, has referred to BA.5 as “the worst variation yet” [11]. Sub-variants BA.4 and BA. 5 are more transmissible than the existing Omicron variant. The percentage of sequences designated BA.4 has grown from less than 1% in January 2022 to more than 35% in some countries [7]. These are continuously evolving strains escaping the antibody protection of vaccinated individuals [12]. Since there have only been a few peer-reviewed scientific pieces of research published on BA.4 and BA.5, their understanding is still limited [7].

Mask mandates have been gradually lifted in many states in the United States, and people have taken off their masks and gotten right back into traveling and socializing, mostly ignoring booster doses [9]. Although it is known that the vaccines are efficient at preventing symptoms of disease, it is not yet known if they also stop the spread of the virus.

The only way to provide herd immunity to the population is to vaccinate everyone [13]. It is necessary to reinstate some of the limitations, including wearing of face masks, greater vaccination and booster shots covering the entire community and restricted social gathering to avoid going back to the stressful times. Each time the virus that causes COVID-19 infects a person again, there are increased health risks [14].

At this time, when the world is coping with a number of crises, including economic and political instability, the Ukraine Crisis, and other Refugee Crisis, we cannot be more carless about COVID-19 to be a pandemic again. Despite the fact that people are giving up on mask mandates, pandemic weariness and the politicization of the virus response, if safeguards are not done, a terrible situation could result.

## Ethical approval

Not applicable.

## Sources of funding

No funding required for the study.

## Author contributions

Jawad Basit: Study conception, write-up, critical review, and, approval of the final version.

Sumia Fatima: Study conception, write-up, critical review, and, approval of the final version.

Usman Ali Akbar: Study conception, write-up, critical review, and, approval of the final version.

Omaima Asif: Study conception, write-up, critical review, and approval of the final version.

Mohammad Ebad ur Rehman: Study conception, write-up, critical review and approval of the final version.

Sajeel Saeed: Study conception, critical review and approval of the final version.

Ka Yiu Lee: Study conception, write-up, and approval of the final version.

## Registration of research studies

1. Name of the registry: Not applicable
2. Unique Identifying number or registration ID: Not applicable
3. Hyperlink to your specific registration (must be publicly accessible and will be checked): Not applicable

## Guarantor

Jawad Basit, Department of Medicine, Rawalpindi Medical University, Block E Satellite Town, Rawalpindi, Pakistan.

Sumia Fatima: Department of Medicine, Rawalpindi Medical University, Block E Satellite Town, Rawalpindi, Pakistan.

## Consent

Not required.

## Declaration of competing interest

All authors declared no conflict of interest.
